# Evidence Based Selection of Commonly Used RT-qPCR Reference Genes for the Analysis of Mouse Skeletal Muscle

**DOI:** 10.1371/journal.pone.0088653

**Published:** 2014-02-11

**Authors:** Kristen C. Thomas, Xi Fiona Zheng, Francia Garces Suarez, Joanna M. Raftery, Kate G. R. Quinlan, Nan Yang, Kathryn N. North, Peter J. Houweling

**Affiliations:** 1 Institute for Neuroscience and Muscle Research, Children’s Hospital at Westmead, Sydney, NSW, Australia; 2 Discipline of Paediatrics and Child Health, Faculty of Medicine, University of Sydney, Sydney, NSW, Australia; 3 Murdoch Childrens Research Institute (MCRI), The Royal Children’s Hospital, Melbourne, VIC, Australia; 4 Department of Paediatrics, Faculty of Medicine, Dentistry and Health Sciences, University of Melbourne, VIC, Australia; Northwestern University, United States of America

## Abstract

The ability to obtain accurate and reproducible data using quantitative real-time Polymerase Chain Reaction (RT-qPCR) is limited by the process of data normalization. The use of ‘housekeeping’ or ‘reference’ genes is the most common technique used to normalize RT-qPCR data. However, commonly used reference genes are often poorly validated and may change as a result of genetic background, environment and experimental intervention. Here we present an analysis of 10 reference genes in mouse skeletal muscle (*Actb, Aldoa, Gapdh, Hprt1, Ppia, Rer1, Rn18s, Rpl27, Rpl41 and Rpl7L1*), which were identified as stable either by microarray or in the literature. Using the MIQE guidelines we compared wild-type (WT) mice across three genetic backgrounds (R129, C57BL/6j and C57BL/10) as well as analyzing the α-actinin-3 knockout (*Actn3* KO) mouse, which is a model of the common null polymorphism (R577X) in human *ACTN3*. Comparing WT mice across three genetic backgrounds, we found that different genes were more tightly regulated in each strain. We have developed a ranked profile of the top performing reference genes in skeletal muscle across these common mouse strains. Interestingly the commonly used reference genes; *Gapdh, Rn18s*, *Hprt1* and *Actb* were not the most stable. Analysis of our experimental variant (*Actn3* KO) also resulted in an altered ranking of reference gene suitability. Furthermore we demonstrate that a poor reference gene results in increased variability in the normalized expression of a gene of interest, and can result in loss of significance. Our data demonstrate that reference genes need to be validated prior to use. For the most accurate normalization, it is important to test several genes and use the geometric mean of at least three of the most stably expressed genes. In the analysis of mouse skeletal muscle, strain and intervention played an important role in selecting the most stable reference genes.

## Introduction

Quantitative real-time PCR (qPCR) is a sensitive fluorescence based technique used to quantify gene transcription and consequently give insight into gene expression and function. The underlying principal of this technique is to enzymatically amplify short sequences using oligonucleotide or probes in a polymerase driven reaction. Several methods exist for the detection of amplified reverse transcriptase (RT) cDNA; the simplest of these involves the incorporation of a fluorescent dye (such as SYBR green) that binds to double stranded DNA, emitting a signal which can be measured in real-time at the end of each RT-qPCR cycle. The point at which cDNA amplification produce a detectable fluorescent signal is termed the cycle threshold (Ct) or quantification cycle (Cq) [1], [2]. The Cq is used to calculate the amount of cDNA - either relatively, by normalizing to a “housekeeper” or “reference” sample or absolutely, by comparing to samples of known concentration and size [3].

A major limiting factor of RT-qPCR is the efficiency of the reverse transcription process and consequently the assumption that the same amount of RNA input results in the generation of the same amount of cDNA [3]–[7]. Reverse transcription efficiency can be affected by many factors including the quality and quantity of initial RNA, oligonucleotide selection (oligo-dT verse random hexemers), and the reverse transcription enzyme chosen [6], [8]. In 2009 the Minimum Information for Publication of Quantitative Real-time PCR Experiments (MIQE) was developed to establish a baseline for all RT-qPCR data reporting [7]. The application of these guidelines enables a more detailed assessment of published RT-qPCR data (including key parameters such as RNA integrity, RT-qPCR quality control and reaction efficiency), while highlighting the importance of validated reference genes.

Selecting suitable reference genes is crucial for data interpretation as results can be significantly skewed by selecting an inappropriate reference gene. Often only one reference gene is utilized for normalizing expression. However, it has been shown that a minimum of three reference genes are required for accurate normalization and that inclusion of further genes may be necessary if the expression differences in the gene of interest are small (i.e. less than 2-fold) [9].

Several programs have been developed to identify the most stable reference genes and consequently a number of methods are now available to assess the reliability of a given gene for RT-qPCR normalization. Most methods utilize a stability value, a measure of variation observed within the samples, to rank each gene, where a lower stability value reflects low variation and therefore appropriateness as a reference gene compared to other candidates. Here we use the latest online reference gene stability assessment tool, *RefFinder* (http://www.leonxie.com/referencegene.php), which ranks the reference genes by assigning weights to each gene based on the geometric mean of four established algorithms; *geNorm* [10], *BestKeeper* [11], *NormFinder* [12] and comparative *delta*-*CT* [13]. This web-based program is freely available and easily accessible, providing a combined analysis of the currently accepted reference gene analysis tools.

To determine a group of suitable reference genes for mouse skeletal muscle analyses, we used the *Actn3* KO mouse model for a case-in-point analysis. The α-actinins are a family of highly conserved actin-binding proteins that belong to the spectrin protein super family [14]. Through evolutionary divergence the α-actinin family has developed considerable functional diversity with a total of four α-actinin isoforms (α-actinin-1 to -4) characterized in mammals. This family can be separated into two broad categories - cytoskeletal calcium-sensitive isoforms (α-actinin-1 and -4) and sarcomeric calcium insensitive isoforms (α-actinin-2 and -3). The sarcomeric α-actinins make up a major component of the skeletal muscle Z-line, which is vital for cytoskeletal organization and muscle contractions. α-Actinin-3 is a highly specialized muscle protein, primarily restricted to fast-muscle fibres. We identified a common polymorphism (R577X) in the *ACTN3* gene of humans which results in the complete loss of ­α-actinin-3 in 16 – 20% of the general population (approximately 1.5 billion people world-wide) [15], [16]. The absence of α-actinin-3 does not result in disease, but has been shown to influence muscle performance in elite athletes [16]-[22] and the general population [23], [24].

In order to understand the role of α-actinin-3 in muscle, we developed an *Actn3* knockout (KO) mouse model which replicates the phenotypes associated with α-actinin-3 deficiency in humans [25], [26]. This mouse model has been successfully established in three different mouse strains; R129, C57BL/6j and C57BL/10, and the phenotypes are robust on all three different genetic backgrounds [27].

To find appropriate genes of interest and reliable reference genes, we utilized a microarray approach to compare the *Actn3* WT and KO mice on the R129 background [28]. The aim of this study was to find suitable reference genes using our microarray data, which we then validated by RT-qPCR in our *Actn3* WT and KO mice across the three different genetic backgrounds (R129, C57BL/6j and C57BL/10). This approach has allowed us to provide a detailed analysis of common and novel reference genes for future mouse skeletal muscle RT-qPCR analyses.

## Materials and Methods

### Samples

This study was carried out in strict accordance with the recommendations in the Australian Code of Practice for the Care and Use of Animals for Scientific Purposes. The protocol was approved by the Children’s Medical Research Institute (CMRI) and Children’s Hospital Westmead (CHW) Animal Care and Ethics Committee (Permit Number: K190). Mice (WT and *Actn3* KO; R129, C57BL6 and C57BL/10) were provided with food and water ad libitum, and maintained on a 12:12 h light and dark cycle. Age matched litter mate controls were used for each strain. Mice were euthanized by cervical dislocation immediately prior to tissue collection. Quadriceps muscles were removed within 30 seconds of euthanasia and placed in RNAlater (Applied Biosystems). All samples were maintained at 4°C overnight before being stored at -20°C prior to RNA extraction for microarray and RT-qPCR analyses.

### RNA isolation and cDNA generation

RNA was isolated from ∼50 mg of mouse quadriceps tissue in 1 mL of TRIzol (Invitrogen) using a TissueLyser (Qiagen) according to the manufacturer’s instructions. The RNA was resuspended into 50 µL DEPC treated water (Bioline). RNA was purified and DNAseI (Qiagen) treated using RNeasy Mini protocol (Qiagen). Quality and quantity was assessed using a 2100 Bioanalyser (Agilent Technologies) 6000 RNA kit (Agilent Technologies) according to the manufacturer’s instructions. One microgram (1 µg) of RNA was reverse transcribed using Super-Script™ III Reverse Transcriptase (Invitrogen), random pdN6 primers (Roche), 0.1 mM DTT (Invitrogen) and 10 mM dNTP (Invitrogen) in a 20 µL volume for 90 minutes at 50°C on a Veriti 96-well thermocycler (Applied Biosystems). All cDNA was diluted 1∶5 in DEPC treated water (Bioline) prior to RT-qPCR. Except for the *Rn18S* and *Gapdh*, the cDNA were diluted to 1∶100 due to the high abundance of these transcripts. Diluted cDNA was aliquoted into working volumes of 10 µl and stored at -80 °C until use. A maximum of three freeze-thaw cycles were performed for each aliquot.

### Microarray and Reference gene selection

As described in Seto *et al*. [28], the quadriceps muscles of six WT and six *Actn3* KO 2-month-old R129 mice were harvested and stored in RNAlater (Applied Biosystems) prior to biotin-labelled aRNA (antisense RNA) synthesis using the MessageAmp II biotin enhanced kits (Ambion) as per manufacturer’s instructions. The concentration of aRNA was determined using a NanoPhotometer (Implen). Reference gene selection were performed following aRNA hybridization to Illumina mouse WG-6 version 1 expression BeadChips, stained with streptavidin-Cy3 conjugate and scanned using the Illumina BeadArray reader. Results were extracted using Illumina BeadStudio software, and the average signal intensities of each reference gene were calculated for each sample and exported to EXCEL. Inter-sample covariance’s (% CV) were calculated for each gene (signal intensity divided by the standard deviation) to determine the degree of variation. Ten reference genes of various expression levels were selected based on either; minimum differences in expression observed in a microarray study and/or commonality of use as reference genes in other studies (Table 1).

**Table 1:** Reference genes selected for this study based on % CV from microarray data.

				Microarray CV (%)	
Rank	Gene ID	Gene Name	Expression Level*	WT only	*Actn3* WT and KO
1	*Rpl41*	ribosomal protein L41	Medium	3.92	3.60
2	*Rer1*	Retention in endoplasmic reticulum 1 protein	Low	4.07	5.60
3	*Actb*	beta actin	Medium	4.75	5.47
4	*Rpl27*	ribosomal protein L27	Medium	5.92	5.89
5	*Rpl7L1*	ribosomal protein L7-like 1	Low	6.39	6.60
6	*Hprt1*	hypoxanthine-guanine phosphoribosyltransferase	Low	8.08	9.78
7	*Ppia*	peptidyl prolyl isomerase A	Medium	10.80	9.90
8	*Aldoa*	aldolase A, fructose-bisphosphate	High	16.16	10.70
9	*Rn18s*	ribosomal 18s RNA	High	11.60	13.82
10	*Gapdh*	glyceraldehyde-3-phosphate dehydrogenase	High	11.75	15.12

Selected reference gene ranks based on WT only and *Actn3* WT verse KO R129 skeletal muscle microarray data. Each rank is based on the coefficient of variance (% CV). * Expression levels were calculated based on the absolute copy number (copies/µL of cDNA) generated following RT-qPCR of R129 skeletal muscle. Low  =  <10^5^; Medium  =  10^5^ – 10^6^; High >10^6^.

### Standard Curve Construction

Primers were designed using primer-BLAST (http://www.ncbi.nlm.nih.gov) (Table S1). For *Aldoa, Hprt1, Ppia, Rn18S, Rer1, Rpl27* and *Rpl7L1* plasmid construction, restriction enzyme sites for *NotI* (ATAAGAATGCGGCCGC) and *ClaI* (CCCATCGAT) were added to the forward and reverse primers respectively (Table S1). The inserts were generated by PCR using Platinum Hi-Fi Taq (Invitrogen) with 20 ng of WT mouse cDNA and cloned into the *NotI/ClaI* sites of pMT3, a version of the pMT2 vector with a modified multiple cloning site. For *ActB* and *Actn2* restriction enzyme sites for *EcoRI* (CGGAATTC) and *SalI* (ACGCGTCGAC) were added to the forward and reverse primers respectively (Table S1). The inserts were generated by PCR using Platinum Hi-Fi Taq (Invitrogen) with 20 ng of WT mouse cDNA and cloned into the *EcoRI/SalI* sites of pGBT9 (Clontech). For *Actn3*, *Gapdh* and *Rpl41* the inserts were generated by PCR using Platinum Taq (Invitrogen) with 20 ng of WT mouse cDNA and cloned into the pCR2.1-TOPO vector (Invitrogen) according to manufacturer’s instructions.

All plasmids were Sanger sequenced (AGRF, Sydney). Plasmid concentrations were calculated based on total plasmid size and concentration as measured spectrophotometrically using a Nanodrop 2000 (Thermo Scientific).

### RT-qPCR

Reverse transcriptase (RT) quantitative real-time PCR (RT-qPCR) was used to measure RNA expression levels of the various reference genes. Primers were designed to span at least one intron boundary using primer-BLAST (http://www.ncbi.nlm.nih.gov) (Table 2). RT-qPCR was carried out on Rotor-Gene 6000 (Qiagen) in a 20 µL reaction volume using, 1μl mouse cDNA (RT process outlined under *RNA isolation and cDNA generation* above), 10 µM of forward and reverse primers, 1x MyTaq buffer (Bioline), 0.2 U MyTaq DNA polymerase (Bioline), 0.4 µL 10x SYBR Green I (Life Technologies) and DNAse-free water. All samples and plasmid DNA were amplified in a minimum of triplicates and a no-template-control (NTC) was included in each reaction. Amplification were performed with a 2 min denaturation step at 95°C, followed by 35 cycles of 95°C for 30 sec, 60–65°C (Table 2) for 30 sec and 72°C for 30 sec. Melt curve analysis was performed from 60–99°C to assess amplification specificity.

**Table 2:** RT-qPCR primers.

Gene ID	Accession Number	Forward Oligo Sequence 5′-3′	Reverse Oligo Sequence 5′-3′	Product Size (bp)	Temp (°C)	Intron Spanning
***Actb***	NM_007393	CCTCCCTGGAGAAGAGCTATG	TTACGGATGTCAACGTCACAC	157	65	Yes
***Actn2***	NM_033268	TGATCCAGAGCTACAGCATCCG	CAGACGCTCATTAGCATGTTGG	161	60	Yes
***Aldoa***	NM_001177307	ACATTGCTGAAGCCCAACAT	ACAGGAAAGTGACCCCAGTG	136	62	Yes
***Gapdh***	NM_008084	AACTTTGGCATTGTGGAAGG	GGATGCAGGGATGATGTTCT	132	65	Yes
***Hprt1***	NM_013556	CAAACTTTGCTTTCCCTGGT	TCTGGCCTGTATCCAACACTTC	100	62	Yes
***Ppia***	NM_008907	GGGTTCCTCCTTTCACAGAA	GATGCCAGGACCTGTATGCT	145	65	Yes
***Rn18s***	NR_003278	GCAATTATTCCCCATGAACG	GGCCTCACTAAACCATCCAA	124	65	N/A
***Rer1***	NM_026395	GCCTTGGGAATTTACCACCT	CTTCGAATGAAGGGACGAAA	137	62	Yes
***Rpl27***	NM_011289	AAGCCGTCATCGTGAAGAACA	CTTGATCTTGGATCGCTTGGC	143	65	Yes
***Rpl41***	NM_018860	GCCATGAGAGCGAAGTGG	CTCCTGCAGGCGTCGTAG	113	65	Yes
***Rpl7l1***	NM_025433	ACGGTGGAGCCTTATGTGAC	TCCGTCAGAGGGACTGTCTT	110	65	Yes

Forward and reverse oligonucleotide sequences 5′ – 3′, product size and annealing temperature.

RT-qPCR amplification efficiency (*E*) was determined for each reaction. The Cq values were determined using a set threshold by the Rotor-Gene 6000 software (version 6), and are defined as the number of cycles needed to reach a specific fluorescent signal threshold of detection. Serial 10-fold dilutions of each plasmid were used to generate a standard curve and amplification efficiencies (E) were determined based on the slope (M) of the log-linear portion of each standard curve (E = 10^-1/M^ -1). The linear dynamic range was determined by the standard curve and correlation coefficients (R^2^) for each gene as reported in Table 3. Finally the limit of detection (LOD) of each reaction was determined based on the lowest/highest standard curve concentration which show a% variance (standard deviation/ mean copies/µl x 100) of <20% (Figure S1; Table S2). Cq values of all samples were within the linear dynamic range for each gene assessed.

**Table 3:** RT-qPCR quality control (R^2^, M and Efficiency) for each reaction.

	WT (R129, C57BL6j and C57BL/10)			*Actn3* R129			*Actn3* C57BL/6j			*Actn3* C57BL/10			*Standard curve – dilution range*
Gene	R^2^	M	Efficiency	R^2^	M	Efficiency	R^2^	M	Efficiency	R^2^	M	Efficiency	Copies/µl
***Actb***	0.9991	–3.469	0.94	0.9914	–3.677	0.87	0.9902	–4.166	0.74	0.9944	–3.530	0.92	9.67e^2^ to 9.67e^6^
***Actn2***	0.9990	–3.852	0.82	0.9852	–4.172	0.74	0.9852	–4.172	0.74	0.9990	–3.852	0.82	9.13e^2^ to 9.13e^6^
***Aldoa***	0.9982	–3.725	0.86	0.9963	–3.463	0.94	0.9852	–3.897	0.81	0.9908	–3.361	0.98	8.45e^4^ to 8.45e^7^
***Gapdh***	0.9995	–3.301	1.01	0.9945	–3.535	0.92	0.9985	–3.510	0.93	0.9928	3.134	1.08	6.86e^2^ to 6.86e^6^
***Hprt1***	0.9975	–3.295	1.01	0.9949	–3.749	0.85	0.9993	–3.546	0.91	0.9932	–3.247	1.03	8.55e^2^ to 8.55e^5^
***Ppia***	0.9910	–3.142	1.08	0.9971	–3.680	0.87	0.9967	–3.587	0.90	0.9852	–3.409	0.96	2.90e^3^ to 2.90e^7^
***Rn18s***	0.9991	–3.233	1.04	0.9991	–3.165	1.07	0.9967	–3.202	1.05	0.9902	–3.325	1.00	3.46e^4^ to 3.46e^7^
***Rer1***	0.9998	–3.328	1.00	0.9998	–3.341	0.99	0.9978	–3.723	0.86	0.9983	–3.491	0.93	9.82e^2^ to 9.82e^6^
***Rpl27***	0.9896	–3.480	0.94	0.9995	–3.514	0.93	0.9962	–3.556	0.91	0.9952	–3.161	1.07	8.98e^2^ to 8.98e^6^
***Rpl41***	0.9994	–2.850	1.24	0.9957	–3.341	0.99	0.9906	–3.337	0.99	0.9890	–3.683	0.87	1.76e^3^ to 1.76e^7^
***Rpl7l1***	0.9984	–3.067	1.12	0.9974	–3.702	0.86	0.9942	–3.287	1.01	0.9835	–3.014	1.15	8.41e^2^ to 8.41e^6^
**Average**	0.997	–3.340	1.01	0.996	–3.576	0.91	0.994	–3.635	0.90	0.992	–2.813	0.98	
**Stdev**	0.004	0.286	0.118	0.004	0.268	0.088	0.005	0.328	0.102	0.005	1.986	0.097	

1. WT mice from R126, C57BL/6j and C57BL/10 mouse strains, 2. R129 WT and *Actn3* KO mice, 3. C57BL/6j WT and *Actn3* KO mice, 4. C57BL/10 WT and *Actn3* KO mice for each selected gene. *R^2^*  =  correlation coefficient (optimal *R^2^*  =  1), *M*  =  the expected average Cq (quantification cycle number) between each 10-fold standard curve dilution (optimal *M*  =  –3.3) and *E*  =  the RT-qPCR reaction efficiency (optimal *E* = 1.00).

### Reference Gene and Statistical Analyses

Raw data obtained using the Rotor-Gene 6000 were exported to Microsoft Excel and further analysed. *RefFinder* (http://www.leonxie.com/referencegene.php) was used to generate stability values for all genes. Statistical analyses were performed using the non-parametric Mann-Whitney U tests for pairwise comparisons of expression data.

## Results and Discussion

In accordance with the MIQE guidelines we have provided an accurate assessment of the RT-qPCR analyses performed in this manuscript. Many factors play a role in experimental RT-qPCR variability and validity. Since the introduction of the MIQE guidelines [7] reporting of these factors has increased, making the assessment of RT-qPCR data more unified. In particular, RNA quality and integrity control, accurate selection of reference genes and RT-qPCR quality control has been shown to significantly impact the reproducibility and efficacy of RT-qPCR experimentation [1], [6], [29]–[31].

### RNA Quality and Integrity

**Figure 1:**
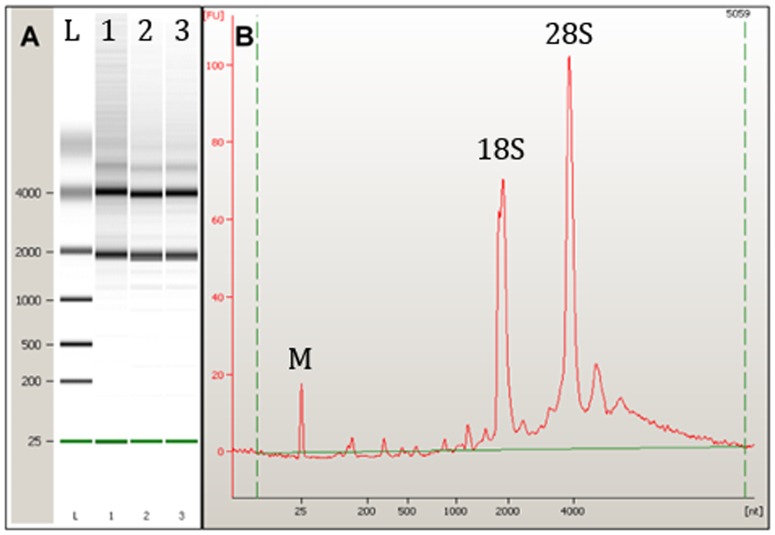
RNA quality control. A) Bioanalyser output of total RNA extracted from mouse skeletal muscle. (L) Ladder, (1 – 3) representative muscle samples. B) Example of Bioanalyser electropherogram of RNA, (M) marker, 18S and 28S ribosomal RNA peaks.

In this study, the RNA extracted from mouse quadriceps were standardized across each strain by utilising qualitative (Nanodrop) and quantitatively (Bioanalysis) RNA analyses prior to cDNA synthesis. Currently the gold standard for RNA quality and quantity control is the use of Agilent’s Bioanalyser [30]. This method utilizes only 1 µL of RNA to give a concentration and objective RNA integrity (RIN) value which reflects the state of degradation for each sample (where a score of 1 is degraded and 10 is intact). The average RIN values obtained from WT and *Actn3* KO quadriceps muscle RNA in this study were 7.67±0.30 (WT n = 6; *Actn3* KO n = 6), 9.14±0.36 (WT n = 6; *Actn3* KO n = 6), and 8.13±0.89 (WT n = 3; *­Actn3* KO n = 5) for the R129, C57BL/6j and C57BL/10 respectively. These RIN values indicate that the samples are adequate for further RT-qPCR analyses (Figure 1).

### Microarray and Reference Gene Selection

Microarray studies are a high throughput method whereby thousands of genes can be assessed in a single experiment to identify differentially expressed transcription pathways. Due to low reliability, poor reproducibility and high false positive rate commonly associated with microarray analysis [32]–[34], it is generally expected that microarray data is validated by RT-qPCR. We have previously published microarray data comparing WT (n = 6) and *Actn3* KO (n = 6) mouse quadriceps samples [28]. Utilising our previously published microarray data we selected ten possible reference genes (Table 1). Genes were ranked by minimum variability based on the coefficient of variance (% CV), where ≤10% is considered to be low variation and therefore represent genes that may be suitable reference genes. Additionally, *Actb, Gapdh, Hprt1* and *Rn18s* were included in our analysis due to their frequent use as reference genes in many studies.

The R129 WT and R129 WT/*Actn3* KO microarray % CV’s were assessed for each potential reference gene. The WT alone analysis shows a% CV range from 3.92 to 11.75. The addition of *Actn3* KO genotypic effect extended this range to 3.6 to 15.12. Although the overall rank of each gene did not change based on *Actn3* genotype, the coefficient of variance increased when taking into account the effect of genotype, suggesting that genotype has an effect on reference gene stability.

### RT-qPCR Quality Control

Prior to RT-qPCR analyses we optimize each of our ten selected reference genes as well as *Actn2* and *Actn3* (our genes of interest) (Table 2). Within every RT-qPCR run, we validated each experiment by including technical replicates of at least four 10-fold serial plasmid DNA dilutions to generate a standard curve. This ensures sample amplification is occurring as expected for each cycle across a range of sample concentrations and enables the calculation of absolute copy number expression values. We utilized measures of technical replicate variability based on the correlation coefficient (optimal R^2^ = 1), the expected average Cq (quantification cycle number) between each 10-fold standard curve dilution (optimal M = –3.3) and the RT-qPCR reaction efficiency (E = 10^−1/M^ − 1) to ensure that each reaction was appropriate for further analysis. As recommended by the MIQE guidelines we have shown these variables for each experiment. Across all experiments we have achieved high correlation coefficients (R^2^), expected Cq (M) and efficiency (E) which suggests that data from these experiments can be interpreted with a high degree of confidence (Table 3).

### Validation of Reference Genes Based on Mouse Strain

**Figure 2:**
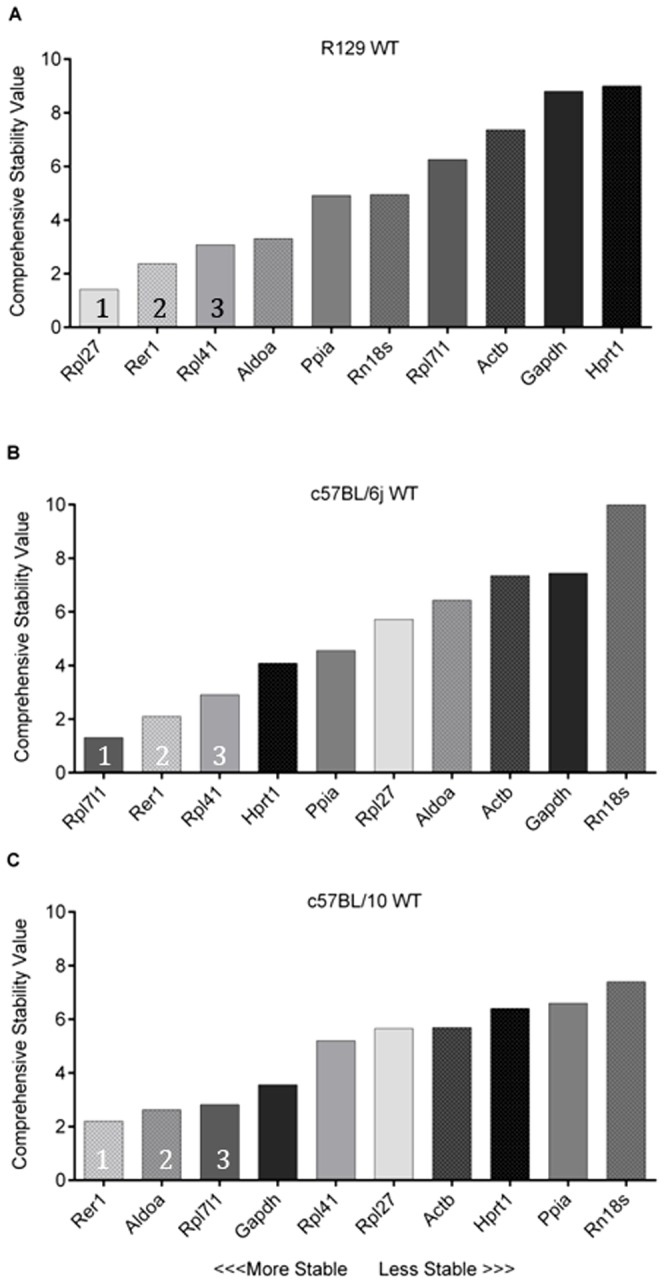
*RefFinder* analysis comparing the selected reference genes for WT quadriceps muscle expression. A) R129 WT, B) C57BL/6j WT, C) C57BL/10 WT. Columns are shaded (light to dark) based on R129 WT gene stability order to represent the shift in gene position between C57BL/6j and C57BL/10 strains. 1 – 3 represent the top three genes in each analysis.

As it is vital to determine the most appropriate or ‘stable’ reference genes prior to RT-qPCR normalization we aimed to identify the best skeletal muscle reference genes in three different mouse strains (R129, C57BL/6j and C57BL/10) without the added complexity of an experimental variant. The ten genes identified by microarray as having low variance in WT mice were validated by RT-qPCR in each of the three different mouse strains (R129 (n = 6), C57BL/6j (n = 6) and C57BL/10 (n = 3)) as shown in Figure 2. Genes such as *Rpl27*, *Rer1* and *Rpl41* that had low variability in the microarray were also shown to be good reference genes by RT-qPCR. *Actb*, however, displayed low variability in our microarray but higher variability by RT-qPCR. Conversely, *Aldoa* which displayed higher variability by microarray had lower variability than many of the other genes when analysed by RT-qPCR. Commonly used reference genes, *Gapdh, Rn18s* and *Hprt1* did not appear to be the most suitable reference genes in R129 mice by RT-qPCR and ranked in the lower half, indicating that there are more stably expressed genes available to use in the R129 mouse strain.

Interestingly, WT mice from the C57BL/6j and C57BL/10 strains did not display the same stability rank for many of the selected genes. The C57BL/6j mice, like the R129 mice, showed higher variability in the commonly used reference genes *Gapdh*, *Actb* and *Rn18s* than the other genes tested. Similar to the R129 mice, *Rer1* and *Rpl41* are highly ranked in the C57BL/6j; however, *Rpl7l1* ranked as the most stable gene in WT mice of this strain. In contrast to the R129 mice, *Hprt1, Rpl27* and *Aldoa* rank intermediately in the C57BL/6j WT mice.

In C57BL/10 WT mice, the genes ranked very differently from both the R129 and C57BL/6j. Notably the comprehensive stability value was more variable between different genes. This is due to a lack of agreement between the different algorithms (*geNorm, BestKeeper, NormFinder* and *DeltaCt*) that *RefFinder* uses to calculate the comprehensive stability value. *Rer1, Aldoa*, *Rpl7l1* and *Gapdh* are all ranked between 2 and 4 and there is no clear gene ranked with a comprehensive stability value of 1. The six least stable genes have comprehensive stability values between 5 and 7.5, representing a failure to clearly differentiate which gene is more stable. This is likely due to the reduced power of the C57BL/6j WT experiment (n = 3).

### Validation of Reference Genes using the *Actn3* KO mouse

**Figure 3:**
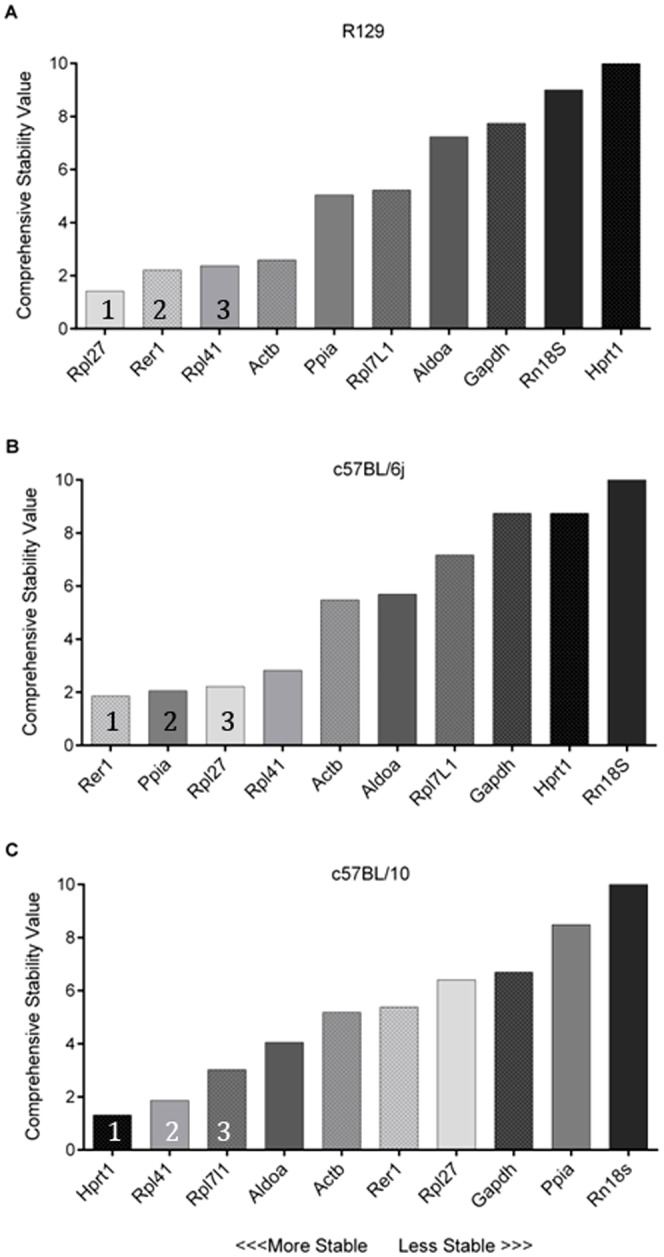
*RefFinder* analysis comparing the selected reference genes in WT and *Actn3* KO mice. A) R129 WT and *Actn3* KO mice, B) C57BL/6j WT and *Actn3* KO mice, C) C57BL/10 WT and *Actn3* KO mice. Columns are shaded light to dark based on R129 gene stability order to represent a shift in position between the C57BL/6j and C57BL/10 strains. 1 – 3 represent the top three genes in each analysis.

To examine how experimental intervention affects the appropriateness of a reference gene, we analysed a common skeletal muscle variant (*Actn3* KO) and looked at reference gene stability between the *Actn3* WT and KO mice across three different mouse strains. The stability of selected genes for each mouse strain (R129, C57BL/6j and C57BL/10) is shown in Figure 3. In this case the stability value is a measure of the effect of α-actinin-3 deficiency on the expression of the reference gene, with lower numbers reflecting a minimal effect highlighting the most stable genes for use with this particular genetic modification.

Comparatively the R129 microarray and R129 RT-qPCR data show similar gene ranking for all reference genes. RT-qPCR results including both the R129 WT (n = 6) and *Actn3* KO (n = 6) mice showed no significant difference in expression between the experimental groups for any genes except for *Hprt1 (*P = 0.025), in agreement with its rank as the least stable gene in this cohort. Results from the microarray and RT-qPCR indicate that the same three genes (*Rpl41*, *Rer1* and *Rpl27*) have the most stable expression in the R129 mice. *Gapdh, Rn18s* and *Hprt1* continue to rank poorly compared to the other reference genes analysed. *Actb* appears to be more stable in this experiment than when comparing only WT mice. This could be due to the increased power of this experiment (n = 12 verse n = 6) or it could be that the addition of a genetic variable affects the stability of other reference genes in this study and hence *Actb* is comparatively less variable under this condition.

The C57BL/6j and R129 data show similar gene rankings with the addition of our genetic variant, *Actn3*, with the same genes ranked in the top and bottom 5. The effect of α-actinin-3 deficiency on the expression of potential reference genes in the C57BL/10 strain differed markedly compared to the R129 and C57BL/6j strains. Genes that are ranked as intermediately stable in the R129 and C57BL/6j such as *Rpl41*, *Rpl7L1* and *Aldoa* appear as the more stable genes (less affected by α-actinin-3 deficiency), while the most stable gene in C57BL/10, *Hprt1*, is one of the least stable in the R129 and C57BL/6j. Interestingly, *Gapdh* and *Rn18s* are still ranked poorly suggesting that other genes might be more suitable reference genes in the C57BL/10 mouse strain.

Among our selected genes, the more commonly used reference genes such as *Gapdh* and *Rn18S* show poor stability values across all mouse strains. However other commonly used reference genes, *Actb* and *Hprt1* are more variable in terms of suitability as reference genes in our cohorts. For example *Hprt1* appears to be the most stable reference gene for the C57BL/10 cohort despite being poorly ranked in both the R129 and C57BL/6j cohorts. While this might be an artefact of the overrepresentation of *Actn3* KO mice in this experiment (WT n = 3, *Actn3* KO n = 5), removal of two *Actn3* KO mice from the analysis does not alter the genes rank (data not shown). Genes that are not traditionally used as reference genes, such as *Rer1* and *Rpl41,* appear to be more suitable as reference genes for all three mouse strains with the addition of our experimental variant. Importantly, there are still notable differences between the rankings obtained for each of the different mouse strains, with all reference genes selected by microarray appearing to alter in suitability depending on strain and experimental intervention. Given that we have selected a genetic variant that is common in the human population, and does not result in disease, it is evident that any experimental intervention results in the need to validate genes before they are applied as reference genes.

### Effect of Reference Gene Selection

**Figure 4:**
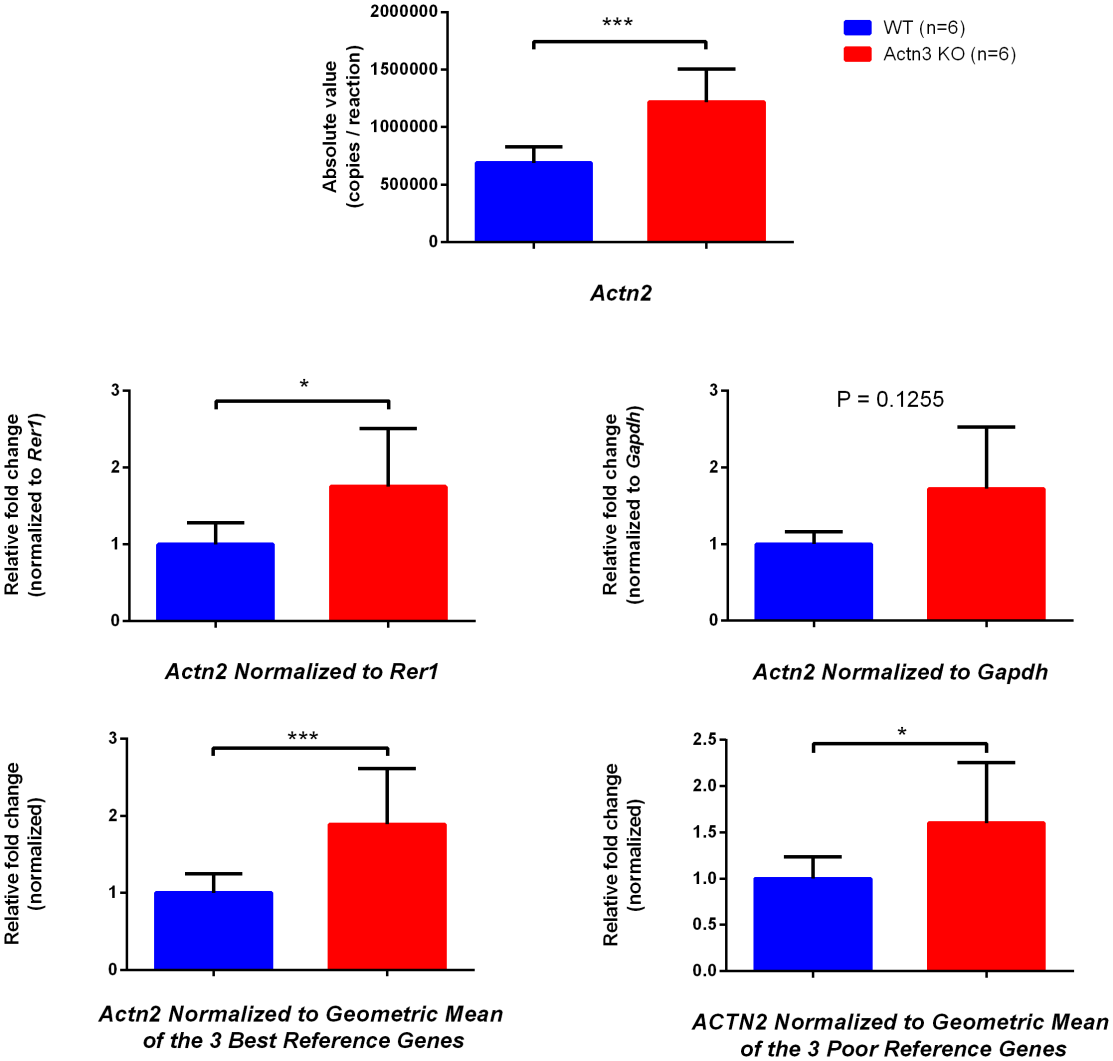
Reference gene selection affects interpretation of a result. *Actn2* mRNA expression is increased approximately two-fold in *Actn3 KO* mouse by Absolute quantification (A). Comparisons of the outcome of normalisation to the best single reference gene (*Rer1*, B), a commonly used but, in this case poor reference gene (*Gapdh*, C) the geometric mean of three top reference genes (*Rer1, Rpl27, Rpl41*, D), and to the three lowest reference genes (*Gapdh, Actb, Rn18s,* E) are shown. (n = 6 WT and n = 6 *Actn3* KO; + StDev; Mann-Whitney U-test *** P = 0.004; * P = 0.026.)

Small differences (i.e. less than 2-fold) have been shown to be hard to quantify by RT-qPCR due to exaggerated or diminished effects of reference gene normalization [9]. A good reference gene is essential to minimize variation due to sample preparation and the reverse transcriptase process. We have previously shown that α-actinin-2 (*Actn2*) is upregulated approximately 2-fold at both the transcript and protein level in *Actn3* KO muscle [28]. We assessed the effect of different reference genes in WT and *Actn3* KO mice and determined their ability to detect differences in *Actn2* mRNA expression in the C57BL/6j strain (Figure S2). By absolute quantification, we see a significant 2-fold upregulation (P = 0.004) (Figure 4A) in the *Actn2* mRNA expression of *Actn3* KO muscle. By normalizing to the top ranked reference gene, *Rer1,* this difference is maintained (P = 0.026) (Figure 4B). However, by normalizing to *Gapdh* (a commonly used reference gene that is less stable in this population), the difference is diminished and significance is lost (P = 0.125) (Figure 4C). As outlined in the MIQE guidelines, normalizing to the geometric mean of the top three reference genes is the most accurate way to assess RT-qPCR data. By normalizing our *Actn2* data to the geometric mean of the three most stable genes (*Rer1, Rpl27* and *Rpl41*, Figure 4D) we are able to maintain the level of significance (P = 0.004) that we see at the absolute quantification level. Normalizing this data to the geometric mean of three less stable reference genes (*Gapdh, Rn18s* and *Actb*, Figure 4E) also shows a significant difference (P = 0.026 ), however the statistical power of this difference is reduced. This data is in agreement with current literature [9], [35]–[37], and shows that utilizing the geometric mean of multiple reference genes is the most accurate way to normalize RT-qPCR data and provides an good assessment of small fold changes.

## Conclusion

The utilization of the MIQE guidelines provides a standardized approach to RT-qPCR analysis – improving reproducibility and the critical analysis of quantitative transcript data. A key factor is the accurate normalization of RT-qPCR results, which is essential for reproducible and accurate quantification of gene expression. We present 10 genes that could be utilized as reference genes for RT-qPCR in skeletal muscle and have shown that the appropriateness of a given reference gene is altered with strain selection and the introduction of a genetic modification (i.e. *Actn3* KO). Within WT muscle the ribosomal genes *Rpl27*, *Rer1* and *Rpl41* ranked in the top 3 of the R129 strain and *Rpl7l1*, *Rer1* and *Rpl41* ranked in the top 3 of the C57BL/6j. *Rer1*, *Aldoa* and *Rpl7l1* were the top 3 ranked genes in the C57BL/10 strain. The commonly used reference genes *Gapdh, Rn18s, Hprt1* and *Actb* were not identified as the best reference genes in any strain. While they appear to be stable in certain circumstances validation under target conditions is require prior to the use of any reference gene. We have shown that reference gene selection is crucial for accurate normalization of a gene of interest (*Actn2*) to ensure a result is reproducible and accurate. Ideally a reference gene should be stably expressed across different tissues, individuals, time points and experimental groups. No single gene has been shown to fit this criterion to date. In agreement with the current literature [9], [35], [36], we recommend normalizing data to the geometric mean of at least three validated reference genes to ensure minimal variation between individuals of an experimental group and the accurate statistical analysis of small fold changes.

## Supporting Information

Figure S1 Representative standard curves from the R129 background analyses of selected reference genes. A minimum of four 10-fold dilutions were performed to generate a standard curve. Standard curves were used to establish correlation coefficient (R^2^), Cq (M) and RT-qPCR efficiency (E) for each reaction, using the Rotor-Gene 6000 software. Exact copy number (copies/µl), standard deviation (Stdev) and % variance (%Var) for each dilution are included in Table S2.
(DOCX)

Figure S2 Representative standard curves for *Actn2* and selected reference genes (*Rer1*, *Gapdh*, *Rpl27*, *Rn18s*, *Rpl41* and *Actb*) in the C57BL6/j genetic background representing the data used to generate results Figure 4.
(DOCX)

Table S1 Reference gene oligonucleotide primer sequences (Forward (F) and reverse (R)), with restriction enzyme sites (bold), product size and vector used to generate Plasmid DNA standard curves.
(DOCX)

Table S2 Calculated reference gene mean copy number (Copies/µl), standard deviation (Stdev) and % Variance (%Var) for each dilution used in the R129 standard curves above (Figure S1). The limit of detection for each gene was determined by the % variance (<20%) or by the highest and lowest dilution, all samples were analysed within the detection range of the each standard curve.
(DOCX)
