# Supplementary figures and images for: Evidence Based Selection of Commonly Used RT-qPCR Reference Genes for the Analysis of Mouse Skeletal Muscle
Source: PLoS One. 2014 Feb 11;9(2):e88653. doi: 10.1371/journal.pone.0088653 (PMC3921188; doi:10.1371/journal.pone.0088653)

**Figure S1**:


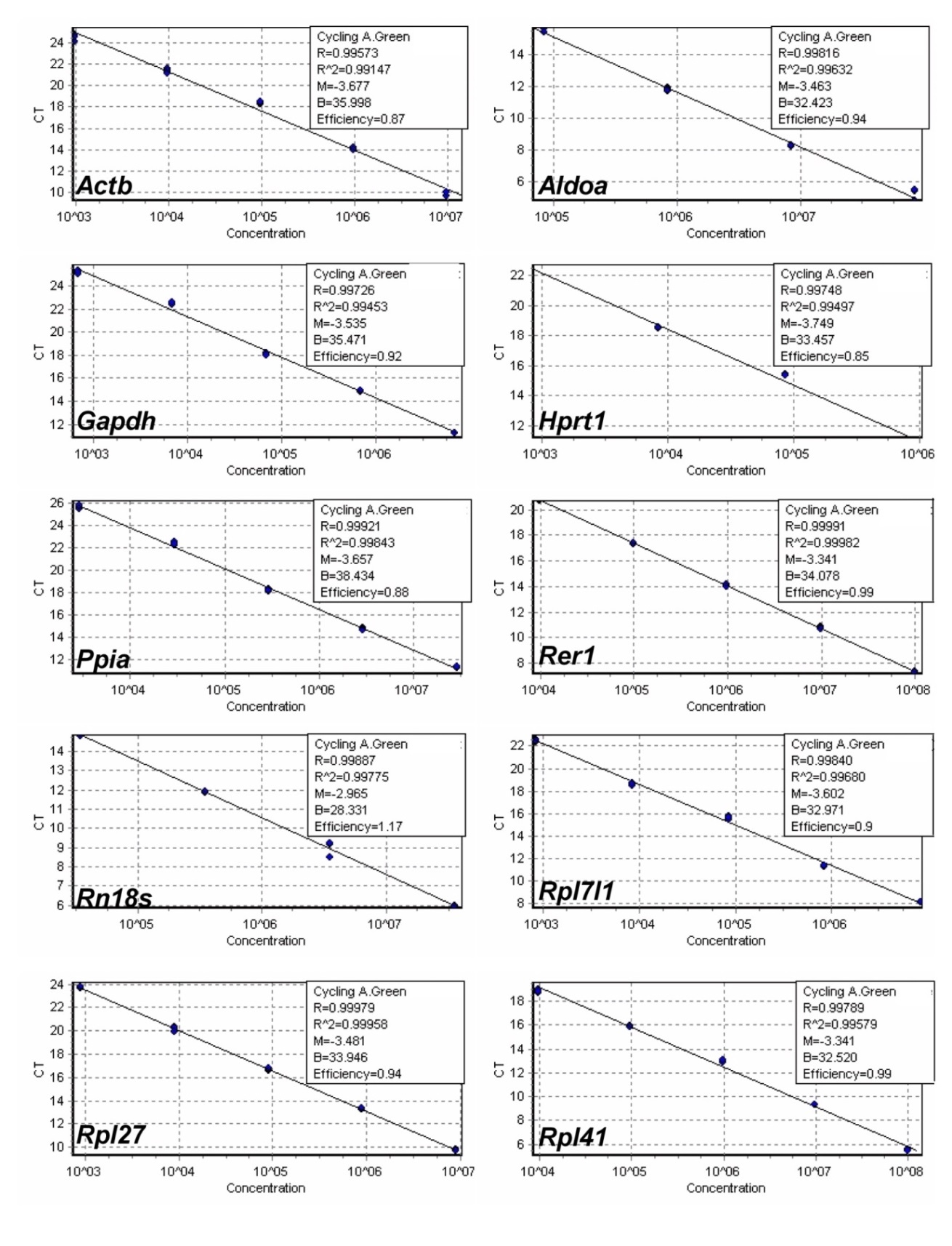

Supplement: Figure S1 — Representative standard curves from the R129 background analyses of selected reference genes. A minimum of four 10-fold dilutions were performed to generate a standard curve. Standard curves were used to establish correlation coefficient (R2), Cq (M) and RT-qPCR efficiency (E) for each reaction, using the Rotor-Gene 6000 software. Exact copy number (copies/µl), standard deviation (Stdev) and % variance (%Var) for each dilution are included in Table S2. (DOCX) [file pone.0088653.s001.docx]

**Figure S2**:


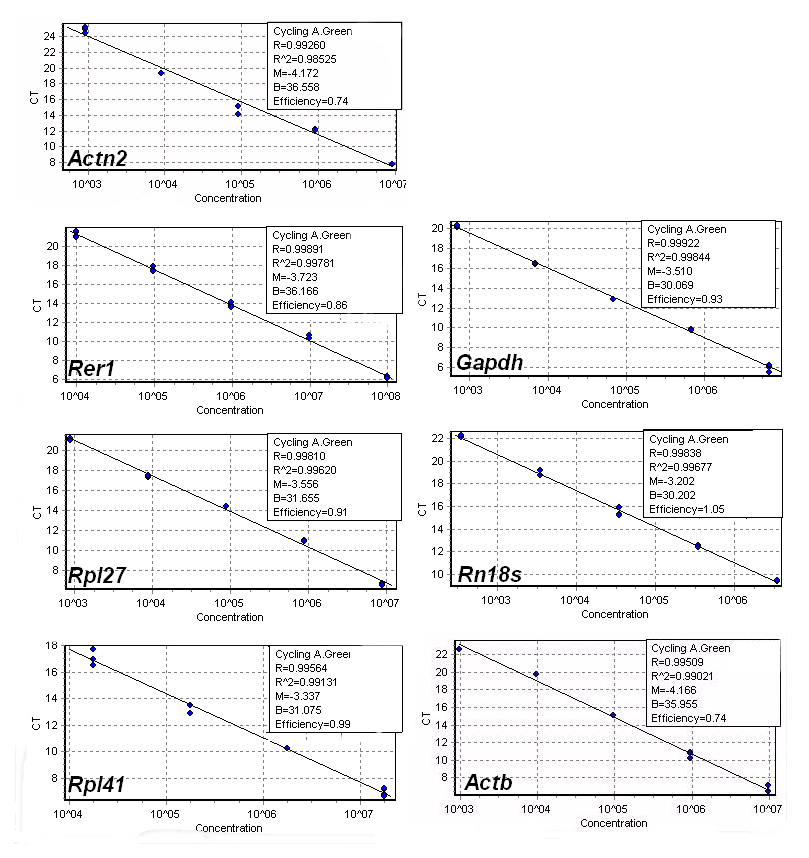

Supplement: Figure S2 — Representative standard curves for Actn2 and selected reference genes (Rer1, Gapdh, Rpl27, Rn18s, Rpl41 and Actb) in the C57BL6/j genetic background representing the data used to generate results Figure 4. (DOCX) [file pone.0088653.s002.docx]
