# Supplementary material for: Evidence Based Selection of Commonly Used RT-qPCR Reference Genes for the Analysis of Mouse Skeletal Muscle
Source: PLoS One. 2014 Feb 11;9(2):e88653. doi: 10.1371/journal.pone.0088653 (PMC3921188; doi:10.1371/journal.pone.0088653)
Supplement: Table S1 — Reference gene oligonucleotide primer sequences (Forward (F) and reverse (R)), with restriction enzyme sites (bold), product size and vector used to generate Plasmid DNA standard curves. (DOCX) [file pone.0088653.s003.docx]

**Table S1**:

| Gene ID | **Accession Number** | **Oligonucleotide Sequence 5’-3’** | **Product Size (bp)** | **Plasmid Vector** |
| --- | --- | --- | --- | --- |
| *Actb* | NM_007393 | F- **CGGAATTCC**CACAGCTGAGGGAAATC  R-**ACGCGTCGACG**CACTGTGTTGGCATAGAGG | 291 | pGBT9 |
| *Actn2* | NM_033268 | F-**CGGAATTCC**AGTCCATCCTGGCCATC  R-**ACGCGTCGACG**AGCGATCTCCTCCATCTTG | 268 | pGBT9 |
| *Aldoa* | NM_001177307 | F-**ATAAGAATGCGGCCGC**AGCAGAATGGCATTGTACCC  R-**CCCATCGAT**GCGCTTGATGTACTCCTCCT | 458 | pMT3 |
| *Gapdh* | NM_008084 | F-GGGTGTGAACCACGACAAAT  R-ACACATTGGGGGTAGGAACA | 323 | pCR2.1-TOPO |
| *Hprt1* | NM_013556 | F-**ATAAGAATGCGGCCGC**TGCTCGAGATGTCATGAAGG  R-**CCCATCGAT**CAAGGGCATATCCAACAACA | 434 | pMT3 |
| *Ppia* | NM_008907 | F-**ATAAGAATGCGGCCGC**GTCTCCTTCGAGCTGTTTGC  R-**CCCATCGAT**TTCACCTTCCCAAAGACCAC | 341 | pMT3 |
| *Rn18s* | NR_003278 | F-**ATAAGAATGCGGCCGC**GTAACCCGTTGAACCCCATT  R-**CCCATCGAT**AGTTCGACCGTCTTCTCAGC | 227 | pMT3 |
| *Rer1* | NM_026395 | F-**ATAAGAATGCGGCCGC**GAAGGGGACAGTGTTGGAGA  R-**CCCATCGAT**TCCTCTTCCCGTGTGTG | 552 | pMT3 |
| *Rpl27* | NM_011289 | F-**ATAAGAATGCGGCCGC**AAGCCGTCATCGTGAAGAAC  R-**CCCATCGAT**TCGCTCCTCAAACTTGACCT | 299 | pMT3 |
| *Rpl41* | NM_018860 | F-TCTTAGCGCCATCTTCCTTG  R-AGAAAGGCGGTCATTTCTCC | 266 | pCR2.1-TOPO |
| *Rpl7l1* | NM_025433 | F-**ATAAGAATGCGGCCGC**GCATGGAAAGGATTGAAGGA  R-**CCCATCGAT**GGAAAGGGCACAGAAATGCC | 354 | pMT3 |
