# Supplementary material for: Evidence Based Selection of Commonly Used RT-qPCR Reference Genes for the Analysis of Mouse Skeletal Muscle
Source: PLoS One. 2014 Feb 11;9(2):e88653. doi: 10.1371/journal.pone.0088653 (PMC3921188; doi:10.1371/journal.pone.0088653)
Supplement: Table S2 — Calculated reference gene mean copy number (Copies/µl), standard deviation (Stdev) and % Variance (%Var) for each dilution used in the R129 standard curves above (Figure S1). The limit of detection for each gene was determined by the % variance (<20%) or by the highest and lowest dilution, all samples were analysed within the detection range of the each standard curve. (DOCX) [file pone.0088653.s004.docx]

**Table S2**:

|  |  |  |  |  |  |  |  |  |  |  |  |  |  |  |  |
| --- | --- | --- | --- | --- | --- | --- | --- | --- | --- | --- | --- | --- | --- | --- | --- |
|  | **1st Point** | | | **2nd Point** | | | **3rd Point** | | | **4th Point** | | | **5th Point** | | |
| **Gene** | **Copies/µL** | **Stdev** | **%Var** | **Copies/µL** | **Stdev** | **%Var** | **Copies/µL** | **Stdev** | **%Var** | **Copies/µL** | **Stdev** | **%Var** | **Copies/µL** | **Stdev** | **%Var** |
| ***Aldoa*** | 84,495,013 | 19829489 | **25.15** | 8,449,501 | 212,519 | 2.24 | 844,950 | 55,788 | 6.27 | 84,495 | 5,718 | 7.43 | NA | NA | NA |
| ***Hprt1*** | 854,508 | 44527 | 4.52 | 85,451 | 2,756 | 4.04 | 8,545 | 1,388 | 15.62 | 855 | 221 | **22.82** | NA | NA | NA |
| ***Rpl27*** | 8,979,902 | 171147 | 1.90 | 897,990 | 31,001 | 3.53 | 89,799 | 6,495 | 7.09 | 8,980 | 1,266 | 13.82 | 898 | 60 | 6.79 |
| ***Rn18s*** | 34,626,508 | 875020 | 2.56 | 3,462,651 | 415,412 | 11.88 | 346,265 | 18,811 | 5.25 | 34,627 | 3,478 | 10.25 | NA | NA | NA |
| ***Gapdh*** | 6,861,279 | 71685 | 1.01 | 686,128 | 28,373 | 4.32 | 68,613 | 4,066 | 4.82 | 6,861 | 351 | 7.79 | 686 | 84 | 9.76 |
| ***Rpl41*** | 17,629,562 | 361109 | 1.64 | 1,762,956 | 22,505 | 1.45 | 176,296 | 6,767 | 5.11 | 17,630 | 330 | 1.92 | 1,763 | 193 | 8.54 |
| ***Rpl7L1*** | 8,410,101 | 74528 | 0.95 | 841,010 | 19,643 | 1.91 | 84,101 | 4,998 | 7.68 | 8,410 | 572 | 6.01 | 841 | 49 | 5.89 |
| ***Ppia*** | 29,046,320 | 969943 | 3.63 | 2,904,632 | 203,236 | 6.57 | 290,463 | 25,216 | 7.37 | 29,046 | 1,611 | 6.54 | 2,905 | 276 | 9.21 |
| ***Actb*** | 9,668,642 | 1432844 | 11.43 | 966,864 | 37,060 | 3.97 | 96,686 | 2,174 | 3.53 | 9,669 | 1,234 | 12.87 | 967 | 314 | **23.09** |
| ***Rer1*** | 9,821,978 | 316298 | 3.13 | 982,198 | 46,330 | 4.96 | 98,220 | 4,449 | 4.47 | 9,822 | 289 | 2.90 | 982 | 35 | 3.59 |
